# Utility of anaerobic bottles for the diagnosis of bloodstream infections

**DOI:** 10.1186/s12879-020-4854-x

**Published:** 2020-02-14

**Authors:** M. Lafaurie, E. d’Anglejan, J. L. Donay, D. Glotz, E. Sarfati, M. Mimoun, M. Legrand, E. Oksenhendler, M. Bagot, S. Valade, B. Bercot, J. M. Molina

**Affiliations:** 1 Department of Infectious Diseases, Saint-Louis Hospital, Paris, University of Paris Diderot Paris 7, Sorbonne Paris Cité, INSERM U941, Paris, France; 2 Department of Infectious Diseases, Saint-Louis Hospital, Assistance Publique des Hôpitaux de Paris, 1 avenue Claude Vellefaux, Paris, 75010 France; 3 Laboratory of Microbiology, Saint-Louis Hospital, Paris, France; 4 Nephrology Unit, Saint-Louis Hospital, Paris, France; 5 Department of Digestive and Emergency Surgery, Saint-Louis Hospital, Paris, France; 6 Department of Plastic Surgery, Saint-Louis Hospital, Paris, France; 7 Department of Anesthesiology, Critical Care and Burn Unit, St-Louis hospital, University Paris Diderot, Paris, France; 8 Department of Clinical Immunopathology, Saint-Louis Hospital, Paris, France; 9 Dermatology Department, Saint-Louis Hospital, Paris, France; 10 Intensive Care Unit, Saint-Louis Hospital, Paris, France

**Keywords:** Blood culture, Bacteremia, Anaerobic

## Abstract

**Background:**

Obligate anaerobes usually account for less than 10% of bacteria recovered from blood cultures (BC). The relevance of routine use of the anaerobic bottle is under debate. The aim of this study was to evaluate the utility of anaerobic bottles for the diagnosis of bloodstream infections (BSI).

**Methods:**

We conducted a 6-month, retrospective, monocentric study in a tertiary hospital. All positive BC were grouped into a single episode of bacteremia when drawn within 7 consecutive days. Bacteremia were classified into contaminants and BSI. Charts of patients with BSI due to obligate anaerobes were studied.

**Results:**

A total of 19,739 blood cultures were collected, 2341 of which (11.9%) were positive. Anaerobic bottles were positive in 1528 (65.3%) of all positive BC but were positive alone (aerobic bottles negative) in 369 (15.8%). Overall 1081 episodes of bacteremia were identified, of which 209 (19.3%) had positive anaerobic bottles alone. The majority 126/209 (60.3%) were contaminants and 83 (39.7%) were BSI. BSI due to facultative anaerobes, obligate aerobes and obligate anaerobes were identified in 67 (80.7%), 3 (3.6%) and 13 (15.7%) of these 83 episodes, respectively. BSI due to obligate anaerobic bacteria were reported in 9 patients with gastro-intestinal disease, in 3 with febrile neutropenia and in 1 burned patient.

**Conclusions:**

Anaerobic bottles contributed to the diagnosis of a significant number of episodes of bacteremia. Isolated bacteria were mostly contaminants and non-obligate anaerobic pathogens. Rare BSI due to obligate anaerobes were reported mainly in patients with gastro-intestinal disorders and during febrile neutropenia.

## Background

The evaluation of patients suspected of having a bloodstream infection (BSI) relies on blood cultures (BC), which identify the bacteria responsible for BSI and assess its antimicrobial susceptibility to guide therapeutic intervention. Standard BC practices in most laboratories are a two-bottle system; one with a vented bottle for aerobes and a second with an unvented bottle for anaerobes. However, many investigators have noted a low yield of anaerobic bacteria with this system with increasing cost and blood drawn [1, 2]. It is argued that the yield of the second unvented bottle could be limited. However, other authors underscore that the anaerobic bottle may be useful to recover obligate anaerobes and may contribute to the prescription of an adequate antibiotic regimen in the case of BSI due to anaerobic bacteria [2–4]. Inappropriate treatment has been shown to impact the clinical outcome of patients with anaerobic related bacteremia, highlighting the importance of identifying anaerobes and studying their antibiotic susceptibility [5]. Indeed, the survival rate of patients with BSI due to strict anaerobes, was significantly worse in patients who received inappropriate therapy compared to those who underwent appropriate therapy [6]. However, study results are inconsistent, which underlines the need for each hospital to determine the combination of bottles that would be most efficient for its patient population [1–4]. Thus, we conducted a 6-month retrospective study to evaluate whether the routine use of anaerobic bottle(s) is useful and in which cases it could lead to the detection of BSI due to obligate anaerobes.

## Methods

This retrospective study involved all hospitalization wards of the Saint-Louis hospital, a 550-bed tertiary hospital with major clinical activities in hematology and oncology.

The study included all BC obtained from consecutive hospitalized patients, aged 18 years or older, during a 6-month period (from the 1st of July, 2014, to the 31st of December, 2014). According to good practice, each BC required the collection of 20 mL of blood per culture set. The nursing staff is supposed to introduce 10 mL of blood in aerobic and anaerobic bottles respectively, and then to send the bottles to the laboratory for processing and bacterial identification. All of the BC sampled during the study period were incubated in a BacT/Alert system with 40 mL of FAN aerobic and anaerobic media. All bottles were placed at 37 °C in the BacT/Alert system for a 5-day incubation period and monitored in accordance with the manufacturer’s recommendations. Bacterial identification was performed using standard procedures. Isolates were identified using the API System (bioMerieux, Marcy l’Etoile, France). Main API identification used API 20E, API and API NH; API 20 Strep and API Coryne; Rapid ID 32A for Gram negative; Gram positive bacteria and anaerobes respectively. Bacterial identification was determined using the online database APIWEB available on the Biomérieux website.

We then determined the number of BC drawn from July 1st to December 31th, 2014, and the number of anaerobic and aerobic isolates.

Positive BC, defined as one positive aerobic bottle and/or one positive anaerobic bottle, were grouped into a single bacteremia episode if they occurred no later than 7 days after the first positive culture with the same organism in the same patient. If another set of cultures was found to be positive more than 7 days after the most recent episode, it was then considered as a separate episode of bacteremia [7].

Bacteremia episodes were segregated into contaminants and BSI. Coagulase-negative staphylococci (CoNS), coryneform bacteria, non-pneumococcal viridans streptococci, *Propionibacterium*, *Bacillus* and Micrococcus species, were considered contaminants when yielded from a unique BC but were considered BSI when yielded from multiples BC and considered clinically significant [8]. Other organisms, such as *Enterobacteriaceae*, *Staphylococcus aureus* or *Pseudomonas aeruginosa*, were defined as pathogens and did require only a single positive BC to be considered as responsible for a BSI [9]. When bacteremia was polymicrobial, each microorganism was considered independently.

A chart review was performed in all patients with BSI due to obligate anaerobes.

The following medical data were collected from patients charts: age, gender, underlying diseases and comorbidities, neutropenia (absolute neutrophil count < 500/mm3), reason for BC sampling, initial and definitive antibiotic treatment and outcome over a 30-day follow-up period.

## Results

**Fig. 1:**
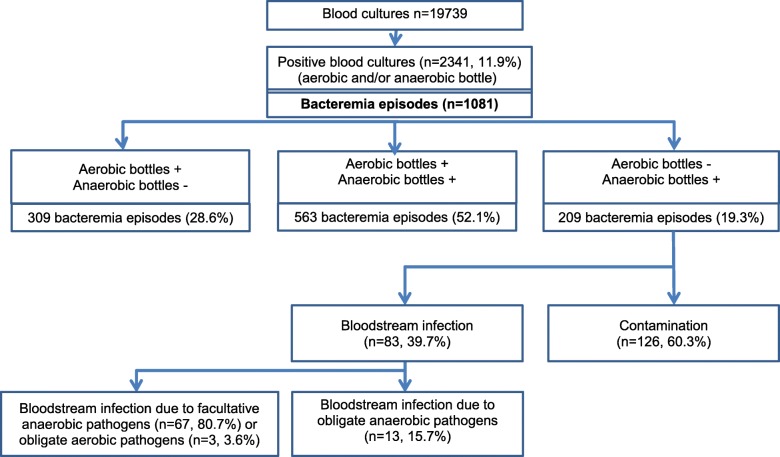
Results of blood cultures during the 6 month-study period, according to aerobic/anaerobic bottles

A total of 19,739 BC were collected during the study period. Overall, 2341 BC (11.9%) were positive (at least one positive bottle, either aerobic and/or anaerobic) (Fig. 1); 1528 anaerobic bottles and 1972 aerobic bottles were positive (7.7 and 10% of BC respectively).

The list of bacteria recovered from BC is shown in Table 1. Facultative anaerobic bacteria were the most frequently isolated bacteria (2020, 86.3%), obligate aerobes and anaerobes were recovered in 292 (12.5%) and 28 (1.2%) of cases respectively. Most positive BC (1159/2341 (48.5%) recovered the same bacteria from both aerobic and anaerobic bottles drawn concomitantly. Aerobic bottles were positive alone (concomitant negative anaerobic bottles in the same set of BC) in 813 (34.7%) cases (Table 1).

**Table 1:** Bacteria recovered from positive blood cultures (one anaerobic positive bottle and/or one positive aerobic bottle)

Bacterial species	Aerobic bottles only (%)	Anaerobic bottles only (%)	Aerobic and anaerobic bottles (%)	Total (%)
**Facultative anaerobic bacteria (48.4)**	**550 (23.5)**	**338 (14.4)**	**1133**	**2020 (86.3)**
Coagulase negative *Staphylococcus*	292 (12.5)	169 (7.3)	517 (22.1)	978 (41.8)
*Staphylococcus aureus*	22 (0.9)	30 (1.1)	118 (5)	170 (7.3)
*Enterococcus spp.*	34 (1.5)	26 (1.3)	60 (2.6)	120 (5.1)
*Streptococcus spp.*	21 (0.9)	18 (0.8)	24 (0.1)	63 (2.7)
*Escherichia coli*	32 (1.4)	32 (1.4)	150 (6.4)	214 (9.1)
*Klebsiella spp.*	16 (0.7)	22 (0.9)	116 (5)	153 (6.5)
Other *Enterobacteriaceae*	25 (1.1)	16 (0.7)	75 (3.2)	116 (5)
Other facultative anaerobic bacteria	108 (4.6)	25 (1.1)	73 (3.1)	206 (8.8)
**Obligate aerobic bacteria (11)**	**263 (11.2)**	**4 (0.1)**	**26**	**292 (12.5)**
*Pseudomonas spp.*	174 (7.3)	4 (0.1)	22 (0.9)	200 (8.5)
*Acinetobacter spp.*	69 (2,9)	0	3 (0.1)	72 (3.1)
Other strict aerobic bacteria	20 (0.9)	0	0	20 (0.9)
**Obligate anaerobic bacteria**	**0**	27* (1.1)	**1 (0.0)**	**28 (1.2)**
Total	813 (34.7)	369 (15.8)	1159 (48.5)	2341 (100)

*Bacteroides fragilis (n = 15), B. thetaiotaomicron (n = 3), Bacteroides. spp. *(n = 1), B. vulgatus (n = 1), Fusobacterium nucleatum (n = 4), F. necrophorum (n = 1), Clostridium sordellii (n = 2)*

Overall, anaerobic bottles led to the identification of bacteria in 1528 (65.3%) BC. But anaerobic bottles were positive alone (aerobic bottles negative in the same BC) in 15.8% of positive BC, identifying mostly facultative anaerobes (338, 14.4%), obligate anaerobes in 27 (1.1%) cases and obligate aerobes in 4 (0.1%) cases.

According to our definition, the 2341 positive BC represented 1081 bacteremia episodes (Fig. 1). Among all episodes, aerobic and anaerobic bottles were both positive in 563 bacteremia (52.1% of episodes), aerobic bottles were positive alone (anaerobic bottles negative during the same episode) in 309 (28.6%) and anaerobic bottles were positive alone in 209 (19.3%) episodes. Of the 209 (19.3%) episodes where anaerobic bottles only were positive, 126 (60.3%) were contaminants and 83 (39.7%) were BSI. Those BSI were due to facultative anaerobes in 67 (80.7%) cases (including one microaerophilic bacteria: *Campylobacter coli*), to obligate aerobes (*P. aeruginosa*) in 3 (3.6%) and to obligate anaerobes in 13 (15.7%). Most frequently isolated facultative anaerobic pathogens were CoNS (7.3%) and *Enterobacteriaceae* (3%) (Table 1).

Of the 13 patients (median age 55 years; range 33–83 years) with BSI due to obligate anaerobic pathogens, 9 (69.2%) were male (Table 2). Strict anaerobic pathogens were *Bacteroides spp*. in 9 cases, *Fusobacterium spp*. in 3 and *Clostridium sordelii* in 1 (Table 2). At the time of BSI diagnosis 6 patients (46.1%) were hospitalized in a surgery ward and 3 (23.1%) in the intensive care unit. In 8 patients (69.2%) BC were performed in a digestive surgery context (acute intra-abdominal infection or surgical site infection). In 3 cases (23.1%), obligate anaerobic pathogens were isolated during an episode offebrile neutropenia following chemotherapy. In 5 patients only (38.5%), the empirical antibiotic treatment had an activity against isolated anaerobic bacteria. In the remaining patients (61.5%), positive BC led to the initiation or a change in antibiotics, according to the microbiological data. Four patients died during follow-up (30 day mortality: 30.8%). Death could be related to anaerobic bacteremia in only 1 patient.

**Table 2:** Characteristics of patients with bloodstream infection due to obligate anaerobic pathogens

Context of blood culture (BC)	Main underlying disease	Pathogen(s) isolated from BC	Other pathogens (site)	Antibiotic treatment before BC results	Treatment modification after BC results	Outcome
Febrile neutropenia	Burkitt’s lymphoma	*Bacteroides* spp.	None	Meropenem and cotrimoxazole	No	Alive
Febrile neutropenia	Acute myeloid leukaemia	*Fusobacterium nucleatum*	None	Piperacilline/tazobactam and spiramycine	No	Alive
Febrile neutropenia	Hodgkin’s lymphoma	*Fusobacterium nucleatum*	None	Cefepime	Metronidazole added	Alive
Acute Diverticulitis	None	*Bacteroides fragilis*	*E. coli* (BC)	Ceftriaxone and ornidazole	No	Alive
Peritonitis mediastinitis, septic shock	Lower oesophageal myotomy	*Bacteroides fragilis*	*P. aeruginosa (urine) and S. maltophilia (sputum)*	Ciprofloxacin and vancomycine	Switch to ornidazole, vancomycine and ceftazidime	Death
Intestinal abscess	None	*Bacteroides fragilis*		Cefotaxime	Metronidazole added	Alive
Surgical site abscess	Oesophageal coloplasty	*Fusobacterium necrophorum*	*Pseudomonas aeruginosa* (abscess)	None	Cefepime initiation	Alive
Rectal abscess	Rectum adenocarcinoma	*Bacteroides thetaiotaomicron*	None	No	Metronidazole added	Alive
Anal abscess	None	*Bacteroides fragilis*	None	Metronidazole and ciprofloxacine	No	Alive
Post-prostate biopsy sepsis	Prostate adenocarcinoma	*Clostridium sordellii,*	*E. coli* (BC) and *E. faecalis*	Ceftriaxone and amikacin	Switch to Piperacilline/tazobactam and gentamicine	Alive
Inhalation pneumonia	Gastric adenocarcinoma	*Bacteroides fragilis*		Piperacilline/tazobactam and amikacin	No	Death
Sacral ulcer	Castelman Disease	*Bacteroides fragilis*	*P. aeruginosa* and *S. aureus* (sputum)	Amikacin and cefepime	Switch to Cefepime, ornidazole and vancomycine	Death
Septic shock	Burn	*Bacteroides vulgatus*	*S. aureus* (BC)	Linezolide	Switch to Piperacilline/tazobactam	Death

## Discussion

Using anaerobic bottles for BC is a standard procedure. However, justification of this practice is still under debate. Some studies claimed that routine use of anaerobic blood cultures is not necessary; proposing that selective rather than routine use of anaerobic culture may be more appropriate [4, 10–14]. However, selection of patients in whom anaerobic blood cultures would be necessary can be difficult [14]. We analyzed herein the efficiency of anaerobic BC in our hospital, in which care of immunosuppressed patients is prominent; we also tried to determine which patients would really benefit from anaerobic BC.

In our study, 11.9% of BC (aerobic and/or anaerobic bottle positive) were positive, which is in the range of previously published studies (6.8 to 11.6%) [15–19]. The number of positive BC using anaerobic bottles (7.7%) was also consistent with previous studies (8.9 to 13%) [14–18] . Interestingly, anaerobic bottles yielded bacteria in 65.3% of positive BC, but were positive alone (without concomitant positive aerobic bottle) in 15.8% of cases.

To better assess the true contribution of anaerobic bottles to the diagnosis of BSI, we grouped all BC sets performed during a single episode of bacteremia. Finally, positivity rates of aero and anaerobic bottles per episode of bacteremia were closed to crude rates of positivity of aero-anaerobic bottles. Rate of positivity of anaerobic bottles within bacteremia episodes was 71.4%. However, in 209 (19.3%) episodes only anaerobic bottles were positive alone. Most anaerobic bottles grew contaminants, 126/209 (60.3%) cases, and only 83 (39.7%) of the 209 positive anaerobic bottles alone were BSI. Contaminations are reported in up to 50% of BC in the literature, CoNS being the most frequently isolated microorganisms, as shown in our study. The high amount of contamination by skin flora in our study may be explained, in part, by a majority of patients having central line, increasing the risk for contamination when blood samples are taken via the catheter [19]. This high rate of contaminants and also the growth of aerobic bacteria in anaerobic bottles may be also interpreted as an evidence for poor sampling procedures.

We also showed that positive anaerobic bottles yielded mostly facultative anaerobic bacteria (338/369, 91.6%) that failed to grow in aerobic conditions (Table 1). This is in line with previous cohort studies demonstrating the utility of anaerobic cultures for the detection of facultative anaerobic bacteremia in adults [20, 21]. Streptococcal species have been reported to grow better with anaerobic bottles [14]. In our study, bacteria growing in anaerobic bottles only were mostly CoNS (7.3% of positive BC) and *Enterobacteriaceae* (3%). Noteworthy, anaerobic bottles also led to the diagnosis of bacteremia related to strict aerobic or microaerophilic bacteria in 4 cases (*P. aeruginosa* in 3, *Campylobacter* in 1).

The improved performance of BC, when anaerobic bottles are used, may be related to the increase volume of collected blood volume and also to the richer medium that may better support bacterial growth. By increasing the volume of collected blood the detection of facultative anaerobic pathogens has been shown to be improved [19]. However, volume of collected blood per bottle could not be evaluated in our study. We could also hypothesize that paradoxically anaerobic bottles led to the identification of aero-anaerobic bacteria because of the involuntary introduction of air inside the bottles. Indeed, strict aerobes were grown from anaerobic bottles in 30 cases (4 cases in anaerobic bottle only, 26 concomitantly in aerobic and anaerobic bottles).

Obligate anaerobes were isolated in only 13 BSI when anaerobic bottles alone were positive (1.2% of all bacteremia episodes). In reports published in the 2000s obligate anaerobes accounted also for only 0.25 to 1.9% of the bacteria recovered in BC [2] [10, 14, 15, 18] [22–24]. The high amount of contamination by skin flora in our study most likely out-competed the growth of any obligate anaerobe in the specimen, explaining in part that few strict anaerobic were isolated. Obligate anaerobes yielded from BC in our study were predominantly *Bacteroides spp.* and *Fusobacterium* spp. (9/13 and 3/13 respectively) in accordance with previous reports [15, 25]. Obligate anaerobic bacteremia have been most frequently related to an abdominal infection source, as well as to pelvic infections, skin and soft-tissue infections [11, 12]. An increased risk for anaerobic BSI has also been reported in patients with cancer and in bone marrow transplant recipients [13, 26], due to chemotherapy-induced neutropenia and disruption of physical barriers [15]. Those data were in agreement with the results of our study. Indeed the source of BSI due to strict anaerobes was the gastro-intestinal tract in 12/13 of our patients (including 3 patients in chemotherapy-induced neutropenia, 2 patients with post-abdominal surgery infection and 1 patient with post biopsy prostatitis) and the skin in 1/13 patient (burned patient). Four patients died despite the initiation of an anti-anaerobes antibiotic prompted by the BC results, underlying the severity of BSI due to obligate anaerobes and the need to use empiric antibiotic therapy active against those pathogens in these patients.

There are some limitations to our study. First, this was a retrospective and monocentric study. Second, the follow-up period was short. Third, the number of positive blood cultures yielding strict anaerobes was limited. Last, the bottles of BC could not been weighed precluding any analyze about the volume of collected blood.

## Conclusions

In summary, this study showed the utility of anaerobic bottles in the diagnosis of 83 episodes of BSI (7.7% of all bacteremia episodes) predominantly due to facultative anaerobes, less frequently to obligate anaerobic pathogens and even to obligate aerobic pathogens. This contribution to BSI diagnosis could be in part related to an increased volume of collected blood, with the anaerobic bottle, and to the involuntary introduction of air and or to the richer medium that may promote bacterial growth. Because of the high number of redundant isolations and low number of positives from anaerobic bottles which represent a very high cost in money, time and effort, better targeting the use of anaerobic bottles may result in substantial savings. At the same time, the laboratory will usually not know the clinical details of patients and clinicians may forget to specifically order anaerobic bottles when appropriate. It seems therefore cautious to keep this procedure of aerobic and anaerobic bottles for BC to optimize the overall detection of BSI, and not only those BSI due to obligate anaerobes.

## Data Availability

The datasets used and/or analysed during the current study are available from the corresponding author on reasonable request.

## Abbreviations

*BC*: Blood cultures
*BSI*: Blood stream infections
CoNS: Coagulase negative *Staphylococcus*
